# Quality of care in family planning services in rural Mozambique with a focus on long acting reversible contraceptives: a cross-sectional survey

**DOI:** 10.1186/s12905-018-0692-z

**Published:** 2018-12-12

**Authors:** Anna Galle, Heleen Vermandere, Sally Griffin, Málica de Melo, Lino Machaieie, Dirk Van Braeckel, Olivier Degomme

**Affiliations:** 1 International Centre for Reproductive Health, Ghent University, Corneel Heymanslaan 10, UZP 114, 9000 Ghent, Belgium; 2 International Centre for Reproductive Health, Rua das Flores no 34, Impasse 1085/87, Maputo, Mozambique

**Keywords:** Family planning services, Long acting reversible contraceptives, Quality of care, Satisfaction, Mozambique

## Abstract

**Background:**

In Mozambique, both the government and partners have undertaken efforts over the last decade to improve FP (family planning) services, especially through training health care providers and promoting the uptake of LARCs (Long Acting Reversible Contraceptives). Despite this, uptake of FP methods has not increased significantly. This study aims to examine women’s knowledge on LARCs, including their main sources of information, and the quality of care of FP services in rural areas.

**Methods:**

We conducted a repeated cross-sectional study, interviewing 417 women leaving FP consultations in 15 health facilities in Maputo Province, Mozambique. The main quality outputs measured were: 1)discussed, preferred and received contraceptive methods, 2)information received on usage and side-effects, 3)client-provider interaction, 4)being informed about the need for a follow-up visit 5)health examinations conducted and travel time to the facility. In addition, knowledge on LARCs was measured among the clients as well as sources of information regarding FP methods. Taking into account the design effect of the study, Chi-square statistics were used to detect differences between groups and linear regression analyses to identify associations between sources of information and higher knowledge.

**Results:**

We found that IUDs (intrauterine devices) and implants were discussed in 23 and 33% of the consultations respectively, but only administered in a very few cases(< 1%). Half of the women were counselled on side-effects of contraceptives; this did not differ between first time clients and follow-up clients. Almost all women(98%) were satisfied with the received service and 83% of the women found the waiting time acceptable. Health examinations were performed on 18% of the women. Overall, women’s knowledge about LARCs was poor and misconceptions are still common. Women who had received FP information through outreach activities had better knowledge than those counselled at a facility.

**Conclusions:**

Our study highlights that only a minority of the women received information regarding LARCs during the consultation and that usage is almost non-existent. Counseling about all types of contraceptives during the consultation is sub-optimal, resulting in poorly informed clients. Multifaceted long-term interventions, focusing on both users and providers, are needed to improve uptake of contraceptives (including LARCs) in rural areas.

**Electronic supplementary material:**

The online version of this article (10.1186/s12905-018-0692-z) contains supplementary material, which is available to authorized users.

## Background

In alignment with global initiatives and the latest evidence, the SDGs (Sustainable Development Goals) include the ambitious target of achieving at least 75% of women of reproductive age (15–49 years) who have their need for FP satisfied with modern methods by 2030 in all countries [1]. Achieving this will require the level of met need for modern methods of contraception to increase by 2.2 percentage points annually between 2014 and 2030 – more than double of today’s average in LMICs (Low and Middle Income Countries) [2].

Although voluntary contraceptive use is rising across most LMICs, in Mozambique progress has been slow. Almost 1 in 4 Mozambican women in a relationship have an unmet need for FP, meaning they are not using contraception despite having an expressed desire to delay, space or limit births [3]. Of all Mozambican women who were married or in a union, 25% used a modern contraceptive in 2015, compared to 17% in 2003 [3, 4]. Furthermore, uptake is highly skewed towards short-acting methods including injectable contraceptives (13% of married women) and oral contraceptives (6%). The use of LARCs (long-acting reversible contraceptives) is very uncommon: in 2015, 1.7% of women used implants and 0.8% IUDs (Intra Uterine Devices) [3].

Long acting reversible contraceptives include the contraceptive implant and IUD [5]. Although there is some debate about whether 3 month contraceptive injections are LARCs, in this paper we do not consider them in this category, in line with the definition used by WHO (World Health Organization) and UNFPA (The United Nations Population Fund) [5]. LARCs have been found to significantly decrease unintended pregnancies and have many advantages compared to other contraceptives: they are easy to use, safe, long-lasting, quickly reversible and 20 times more effective than combined oral contraceptives [6, 7]. As a result, the WHO recommends both implants and IUDs for women with or without children of any age, including adolescents and women over 40 [8].

Major barriers to LARCs uptake by women can be clustered under three main categories: 1) User-related, 2) provider-related, and 3) cost-related [9]. Although these barriers exist worldwide, some are more problematic in LMICs, and additional barriers related to context and culture may arise. Firstly, on the user side, a lack of awareness, fear of side-effects and misconceptions about LARCs can hamper uptake particularly in rural areas [10]. Secondly, on the provider side, Mozambique is dealing with weaknesses in the supply management system, inadequate infrastructure and insufficiently trained health care providers which hampers universal access to contraceptives in general [11]. Misconceptions on the provider side (such as reluctance to offer LARCs to young, unmarried women) can limit the usage of LARCs. Providers often worry about whether LARCs are safe for young, unmarried women and if the use of LARCs could affect their fertility in the future [12]. Finally, cost is in principle not an important factor in Mozambique since FP services are free for patients in public facilities; however, informal payments are common, as well as indirect costs associated with accessing health care such as transport. Informal or under the table payments to health service providers have been estimated to constitute between 10 and 45% of total out-of-pocket expenditure for healthcare in many low-income countries [13]. Informal payments in poor resource settings are mainly made in order to get priority in the waiting line or receive better quality of services [13, 14].

Various efforts have been undertaken to strengthen the health system and remove barriers to FP services in Mozambique [15]. The national government and its development partners have been engaged in improving FP services in the region through developing guidelines, training health care providers, introducing new modern methods (such as implants in 2012 [16]) and the integration of FP in other health services. In addition, Mozambique is one of the priority countries of the Family Planning 2020 (FP2020) Initiative [17, 18], a global partnership that supports the rights of women and girls to decide, freely, and for themselves, whether, when, and how many children they want to have. The Mozambican government signed a commitment agreement in light of FP2020 to increase access to long-acting and permanent methods from 1% to 5% of women by 2015 and to increase the contraceptive prevalence rate from 12% in 2008 to 34% in 2020 [18, 19].

While important steps have been taken, it is not clear to what extent these efforts have actually improved the quality of care in FP services in rural areas in Mozambique, which is essential to ensure adequate uptake of contraceptives (i.e. attracting new contraceptive users and retaining existing users) [20]. Quality of care is a multidimensional issue that can be defined and measured in various ways depending on the stakeholder’s interest [21]. The Bruce-Jain framework [22], developed in 1990, is often considered the central paradigm for quality of care in FP services [21, 23, 24]. It defines quality of care as “the way individuals and clients are treated by the system providing services” and puts forward six essential elements of quality of care: choice of methods; information given to clients; technical competency of providers; interpersonal relations; follow-up mechanisms; and appropriate constellation of services. All elements, except for technical competence, have several indicators that can be assessed through exit interviews with family planning clients [25].

### Objectives

We wanted to assess quality of care in family planning services in rural Mozambique focusing on outcome indicators relating to 5 of the 6 elements of the Bruce-Jain framework: 1) discussed, preferred and received methods, 2) received information on usage and side-effects, 3) client provider interaction, 4) informed about the need for follow up, 5) general health examinations conducted and travel time. As a secondary objective we examined the importance of health care facilities as a source of information on FP methods by investigating the association between women’s knowledge of LARCs and information sources.

## Methods

### Setting

In Mozambique, primary health care facilities at district level can be divided into type I and type II health centers and health posts. According to guidelines of the National Ministry of Health, type II facilities should offer male and female condoms, oral contraceptives, 3 month injectable contraceptives, implants, and IUDs [26]. Implants were only recently introduced in Mozambique, in mid-2012 [16]. In our study, we focused on type II health care centers in two districts (Manhiça and Marracuene) in Maputo province, Mozambique. We only included type II health facilities because they encompass provision of a range of family planning services, while health posts usually do not, and type I health centers serve as referral centers. Although we originally intended to include all 21 type II facilities located in those districts, we excluded 6 of them due to: being closely linked to a type I health facility and serving as a referral center (2), being extremely hard to reach (2) or not providing FP services (2).

In March 2015 the Maputo provincial health department (DPS - Direcção Provincial de Saúde), in collaboration with ICRH (International Centre for Reproductive Health), organized refresher training for the staff from the participating health centers on provision of FP services, so as to ensure all health centers could provide all methods. During three full days, all available modern contraceptives (male and female condom, combined oral contraceptives, injections, implant, and IUD) were discussed and practical sessions were organized, focusing on insertion of implants and IUDs. Training also included inter-personal communication skills and FP counseling. In addition, all health centers received the necessary equipment to provide all methods, if needed. Afterwards, all health centers participated in a project aiming at improving stock management of contraceptives (April 2015 until February 2016). Monthly visits were conducted by ICRH in order to monitor improvements in supply management. In addition, providers’ motivation was measured 3 times, with 4 month intervals. The results of this project are published elsewhere [27].

### Instruments

The outcome indicators used for each element of the Bruce-Jain framework for quality of care were based on the work of Strobino et al. (2000) [25] and completed with some additional quality indicators based on more recent literature [28, 29]. The aspect of choice of methods was assessed by asking which methods were discussed, which method was given, whether or not the client received her chosen method and was satisfied with the given method. Indicators related to information given to clients include having received verbal and/or written information about how to use their method and about its side effects and having received any material about FP such as a brochure, pamphlet or booklet. Interpersonal relations focuses on the client-provider interaction and included indicators related to treating the client with respect, feeling comfortable and general satisfaction. Continuity and follow-up indicators include whether or not she was informed about the need for follow up and where to go in case of emergency. The domain of appropriate constellation of services included whether or not the client was examined, travel time to the facility and opinion regarding the waiting time and opening hour. Health examinations included blood pressure measurement, weight monitoring and testing for HIV and STIs (Sexually Transmitted Infections). The questionnaire can be found in the supplementary files (see Additional file 1).

Knowledge about LARCs was measured among the clients by four multiple choice questions (yes / no / don’t know) (Fig. 2), which were based on research conducted by Pathfinder and were adapted to the local context based on input of ten local experts [30]. Answers were recoded as “0 = wrong answer”, “1= I don’t know”, “2 = correct answer” and a total score was calculated ranging from 0 to 8.

### Data collection

Data collection took place in three rounds, in June 2015, October 2015 and March 2016 (rounds 1, 2 and 3 respectively); the repeated study design was part of the study regarding stock management published elsewhere [31]. Due to this design we checked for change over time as we expected to find a high number of women satisfied about the received care (due to the trainings and supervision) at the beginning of the study and a fade out effect after time. We collected 417 exit-interviews of women exiting FP consultations. For each round, two fieldworkers spent two mornings at each health center during which they invited every woman exiting the FP consultation for an interview.

The administration of the questionnaire took place in a non-clinical environment outside the facility, and lasted for approximately 15 min. Confidentiality was guaranteed at all times and no names were asked. Before the start of the interview, all women received information regarding the content and objective of the questionnaire, after which written consent was obtained. The interview was pen-and-paper administered and data was entered in Epi-info. Data cleaning and analysis were conducted in R.

### Sample size

Prior sample size calculation was done based on the indicator of general satisfaction included in the survey. We used an online sample size calculator to estimate the required sample size to detect dissatisfaction among 10% of the female FP clients regarding FP methods (http://www.select-statistics.co.uk/sample-size-calculator-proportion). The estimates used can be found in Table 1 and resulted in a sample size of 136 for each round of data collection.

**Table 1:** Sample Size calculation

Socio Demographic Data	Manhiça & Marracuene Districts
Population^a^	242.617
Women^a^	130.017
Women of reproductive age (national 41.8%^b^)	54.347
Using contraceptives (national 12.1%^b^)	6576
Sample for 10% dissatisfaction rate	136

^a^data from DPS

^b^based on data from DHS 2011

### Data analysis

#### Post-sampling weight analysis

After data collection, we noticed that the distribution of participants sampled per health facility did not reflect the distribution of patients we expected (based on records of patients in 2014). Due to closed facilities on the day of the visit, distance and time constraints some health facilities were overrepresented and others underrepresented. To account for this, the relative weight for each health facility within the sample was calculated. That is, observations more likely to be selected (e.g., from oversampling) received a smaller weight than observations less likely to be selected. Subsequently, raw weights for each health center according to the population size were calculated based on the female FP clients each facility received in 2014. Sample weights were then obtained by dividing the raw weights by the relative weights. Sample weights were then applied in the computation of statistics from the sample observations [32]. Finally, we also took into account the design effect by using the *survey* package in R (*svydesign*) and adjusted for health centers weight and clustering effect for all further analysis [32–34]. The Design Effect for our outcome measure (general satisfaction) was calculated and considered as acceptable (DEFF 1.25).

#### PCA (principal component analysis)

We performed a Principal Component analysis on the four questions related to knowledge about IUDs and implants in order to examine the dimensionality of the data and detect the correlation between variables. (see Additional file 2). For computing PCA we took into account the assumptions of Hatcher & Stepanski (1994) [35]: Interval-level measurement, Random sampling, Linearity and Normal distributions of the variables were respected. Both biplot and a scree plot were performed. Subsequently a reduced set of components was extracted from the knowledge variables.

#### Chi-square statistic & linear regression analysis

Simple descriptive analysis was done to explore sociodemographic characteristics of the population and outcome indicators for quality of care. Pearson chi-squared statistics were calculated to assess whether there was a significant difference in the percentages of women that were satisfied between rounds and main sources of FP information between the rounds. Also for detecting a difference in consultation content between first time users and follow-up clients Pearson chi-squared statistics were used.

We examined significant predictors for knowledge of LARCs by building a generalized linear model with inverse-probability weighting and design-based standard errors in R. Assumptions concerning the data structure were verified graphically in R. A linear model was built with the principal component of knowledge as continuous outcome variable and sources of information as dichotomous predictors. The selection of the model was done in different steps. First, we selected different predictors for knowledge based on the literature (such as age, marital status, travel time to the facility and sources of health information). The main sources of information were grouped into four categories: 1) health promotion in the clinic, 2) radio or television, 3) community talks, activists or community meetings, 4) mobile teams or community health workers. These categories were renamed as 1) health promotion 2) Mass Media 3) community campaigns and 4) outreach activities. Mobile teams and community health workers were put in one category as they are both part of the national health service outreach activities. Activists were classified under community campaigns as their activities are mostly linked to community meetings and community talks. We built eight different models by adding and reducing the number of predictors and compared these models. This was in order to reduce the risk of over or under-fitting a model, which may not capture the true nature of the variability in the outcome variable [36]. Finally, we utilized AIC (Akaike’s Information Criterion) to select the best model, which is the model with the lowest AIC score.

## Results

### Sociodemographic characteristics

Local fieldworkers approached 422 women of which 5 refused to participate due to time constraints. In total 417 women were interviewed and included in the sample, 43% of the interviews took place at health centers in Manhiça and 57% in Marracuene. Both weighted and unweighted frequencies of the sociodemographic characteristics are given in Table 2. Around 62% of the women were aged between 21 and 35 years and the women had an average age of 28 years old. The majority (83%) of women were in a relationship.

**Table 2:** Sociodemographic characteristics

	n	Unweighted %	Weighted %
Sociodemographic Characteristics			
District			
Manhiça	(*n* = 178)	42.89	62.00
Marracuene	(*n* = 237)	57.11	38.00
Marital Status			
In a relationship	(*n* = 339)	82.89	84.77
Single	(*n* = 70)	17.11	15.23
Age			
<= 21	(*n* = 75)	18.25	19.49
> 21 & < = 25	(*n* = 83)	20.19	25.81
> 25 & < = 35	(*n* = 175)	42.58	35.90
> 35 years	(*n* = 78)	18.98	18.80
Awareness of Family Planning			
Heard about FP before the consultation			
Yes	(*n* = 394)	96.33	96.82
No	(*n* = 15)	3.67	3.18
Received FP information in the last 3 months			
Yes	(*n* = 294)	75.00	67.86
No	(*n* = 98)	25.00	32.14

### Choice of method

**Fig. 1:**
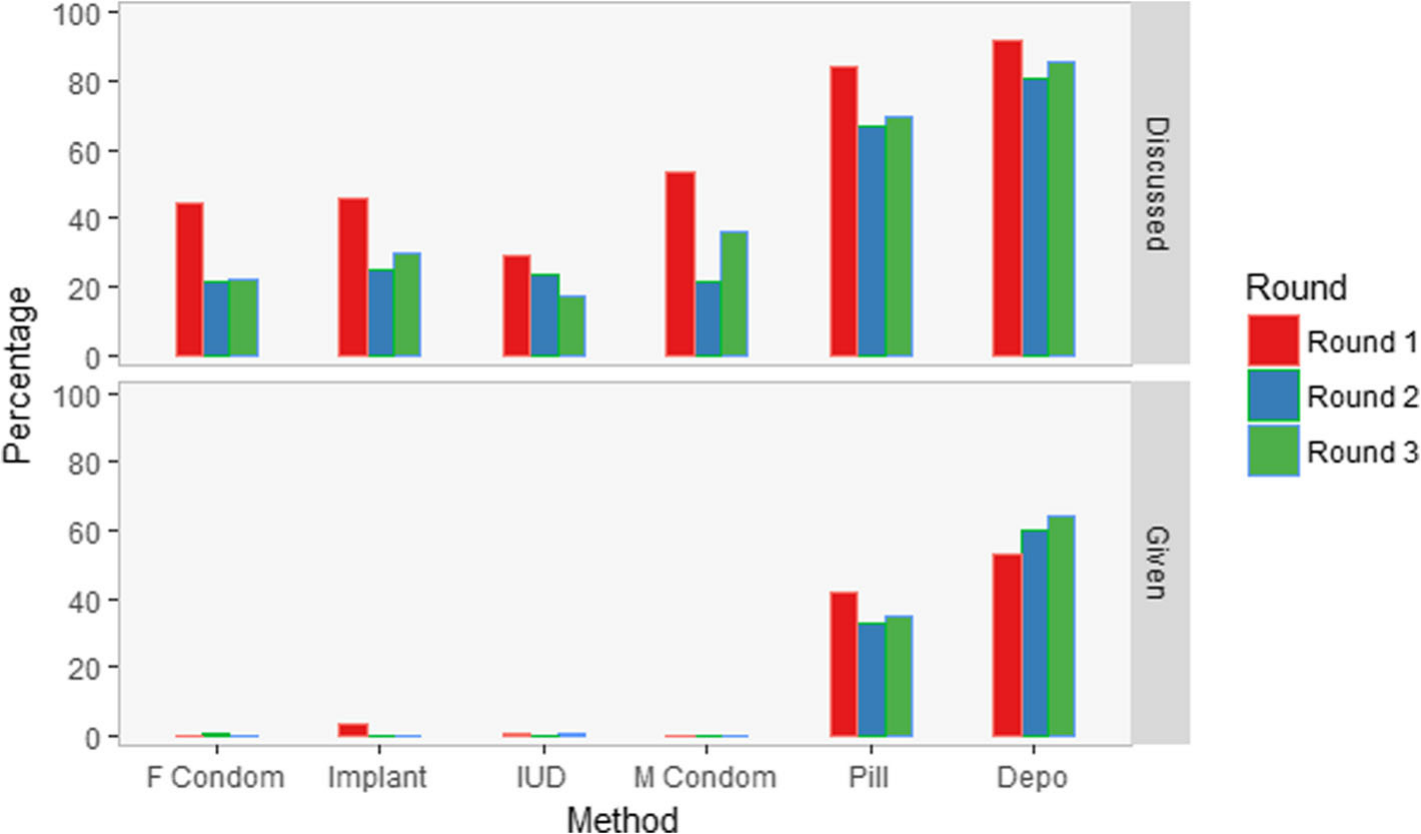
Percentage of women who received information regarding the method during consultation and percentage of women who received the method during consultation per round. Pill = Oral Contraceptives. Depo = Injectable Contraceptives

Women were asked which methods were discussed during consultation (Fig. 1). The number of methods discussed varied from 0 to 6, with an average of 2 methods, and did not differ between first time users or follow-up visits (*t* = 0.853, *p* = 0.41). Injectable (=Depo) and oral contraceptives (=Pill) were discussed most frequently (Fig. 1).

Women were also asked which method they received (Fig. 1). Implants were only given in round 1 (5 times) and IUDs only once in round one and once in round three. Implant dropped from 5 times given in round one to not given at all in round two and three. IUD was given once in round one and once in round three. Female condom was only given in round two and male condom once in round one and once in round two.

Five percent of the women did not receive the method they preferred. A third of women who did not receive their preferred method mentioned that the method was not available in the health center at that time. Three percent of the women were not satisfied with the method received (Table 3).

**Table 3:** Choice of methods, interpersonal relations, follow -up and continuity, and constellation of services

	n	Unweighted %	Weighted %
Choice of Method			
Received preferred method			
Yes	(*n* = 386)	94.84	94.56
No	(*n* = 21)	5.16	5.44
Why not received			
Don’t know	(*n* = 1)	4.76	9.69
Not available	(*n* = 7)	33.33	25.84
Not recommended by provider	(*n* = 5)	23.81	18.04
Other reasons	(*n* = 8)	38.10	46.43
Satisfied with the method received			
Yes	(*n* = 399)	97.32	97.50
No	(*n* = 11)	2.68	2.50
Interpersonal Relations			
Satisfied in general			
Yes	(*n* = 398)	97.79	98.20
No	(*n* = 9)	2.21	1.80
Treated with respect			
Yes	(*n* = 411)	99.76	99.48
No	(*n* = 1)	0.24	0.52
Felt comfortable			
Yes	(*n* = 408)	99.03	99.11
No	(*n* = 4)	0.97	0.89
Would you recommend the service			
Don’t know	(*n* = 2)	0.49	0.75
Don’t recommend	(*n* = 1)	0.24	5.88
Recommend moderately	(*n* = 67)	16.34	18.55
Highly recommend	(*n* = 340)	82.93	74.82
Follow-up and Continuity			
Informed where to go in case of problems			
Yes	(*n* = 282)	68.61	72.41
No	(*n* = 129)	31.39	27.59
Informed about follow-up visit			
Yes	(*n* = 407)	99.02	98.53
No	(*n* = 4)	0.98	1.47
Constellation of Services			
Transport to health facility			
On foot	(*n* = 299)	72.57	81.44
Minibus	(*n* = 111)	26.94	15.35
Others	(*n* = 2)	0.49	3.20
Travel time to the facility			
< 15 min	(*n* = 121)	29.88	32.71
15-30 min	(*n* = 130)	32.10	34.52
30-60 min	(*n* = 115)	28.40	19.74
> 60 min	(*n* = 39)	9.63	13.03
Waiting time acceptable			
Yes	(*n* = 321)	77.91	82.70
No	(*n* = 91)	22.09	17.30
Convenient opening hours			
Yes	(*n* = 404)	98.78	99.23
No	(*n* = 5)	1.22	0.07
Health exams conducted			
Yes	(*n* = 46)	11.20	11.36
No	(*n* = 365)	88.80	88.64

### Information given to clients

For 24% of the women it was the first time they received the given method (Table 4). Information about the usage of the method was given to 88% of the new clients and 84% of the follow-up clients. Potential side effects were discussed with 50% of the new users and to 47% of the women who came for a follow-up visit. Information about where to go in case of problems was given to 68% of the new clients and 72% of the follow-up clients. Material about FP was given to 2% of the new clients and 6% of the follow-up users. There was no significant difference between first time clients and follow-up clients regarding the content of the consultation (Table 4).

**Table 4:** Information given to clients

Information	n	Weighted %	Weighted %	X^2^ Test of independence
Type of Consultation	(*n* = 411)	First Time 24.03	Follow-up 75.97	
Information about usage				
Yes	(*n* = 329)	88.23	83.60	X^2^ = 1.2688 *P* = 0.2176
No	(*n* = 80)	11.77	16.40	
Information potential side effects				
Yes	(*n* = 168)	50.30	46.96	X^2^ = 0.3452 *P* = 0.6899
No	(*n* = 239)	49.70	53.04	
Received any material about FP				
Yes	(*n* = 18)	2.02	5.64	X^2^ = 1.983
No	(*n* = 391)	97.98	93.36	*P* = 0.1578

### Interpersonal relations

General satisfaction was very high (98%) and 75% of the women would highly recommend the service to a friend/relative (Table 3). Almost all women reported that they felt they were treated with respect (99%) and felt comfortable (99%). Percentage of women satisfied didn’t differ between round 1, 2 or 3 (X^2^ = 1.72,*p*-value = 0.51).

### Mechanisms for continuity and follow up

Almost all women were informed about a follow-up visit (Table 3). No difference was found between new users and follow-up visits (X^2^ = 1.96, p-value = 0.54). One third of the women were not told where to go in case of problems or emergencies (Table 3). Again no difference was found between new users and follow-up visits regarding the percentage of women told where to in case of problems or emergencies (X^2^ = 0.48, *P* = 0.45).

### Constellation of services

Health examinations were performed on 17% of the new users and 11% of the follow-up visits, but the difference between new users and follow-up visits was not significant (X^2^ = 2.88, p-value = 0.17). Overall 12% of the women were examined during the FP visit. Forty-three out of 46 women also received information (93%) regarding the health exams that were performed.

Around 73% (*n* = 299) of the women had walked to the health care center, taking on average 45 min to reach the clinic. For 83% (*n* = 321) of the women the waiting time inside the clinic was acceptable (Table 3) and almost all women (99%) were satisfied with the opening hours of the health center.

### Knowledge about LARCs and sources of information

**Fig. 2:**
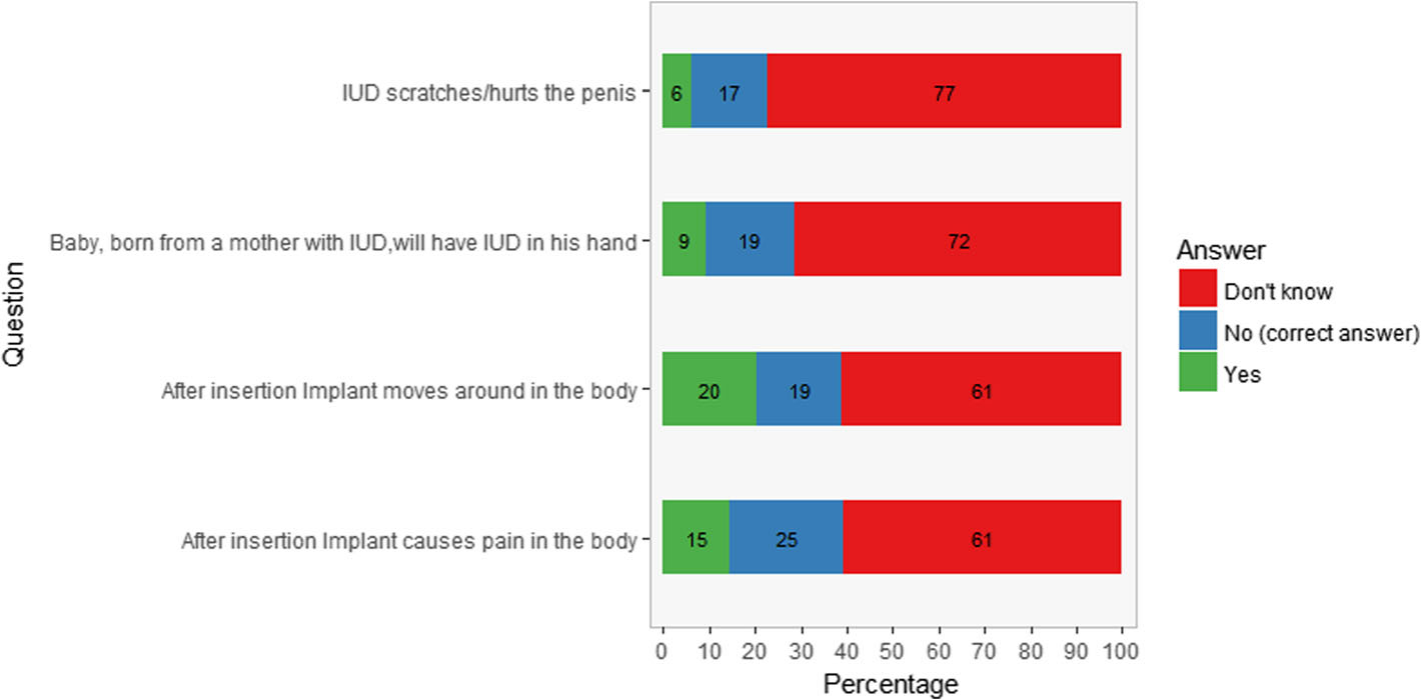
Knowledge of IUDs and Implants among female FP clients

Almost all women had heard about FP before the consultation (97%) and 68% had received information regarding FP in the last 3 months (Table 2). Knowledge regarding IUD and implants is limited, women answering correctly ranged between 14 and 22% among the four questions (Fig. 2). Thirteen percent (Unweighted *n* = 63) of the women had never heard about implants and 15% (Unweighted *n* = 74) had never heard about IUDs. Of the women who had heard about implants or IUDs, more than half of them did not know the answer to the knowledge questions regarding these methods (Fig. 2).

A PCA analysis was conducted on the four knowledge questions. One main PCA component, reflecting overall knowledge, could be identified based on interpretation of the scree plot and criteria proposed by Holland et al. (2008) [33]: 1) Ignore principal components (PCs) at the point which the next PCA offers little increase in the total variance explained and 2) include all those PCs up to a predetermined variance explained, where we considered 80% as an acceptable threshold. The biplot (see Additional file 2) reflected the structure of the data: questions related to IUD and questions related to implant (F2 & F3) each pointed in a different direction of the x-axis. All PCA loadings (=the covariance/correlations between the original knowledge questions and the unit-scaled components) were close to 0.5. PC1 was plotted against the total knowledge score (ranging from 0 to 8) (see Additional file 2). Based on the loadings shown in Additional file 2, we labeled Component 1 as overall knowledge.

**Fig. 3:**
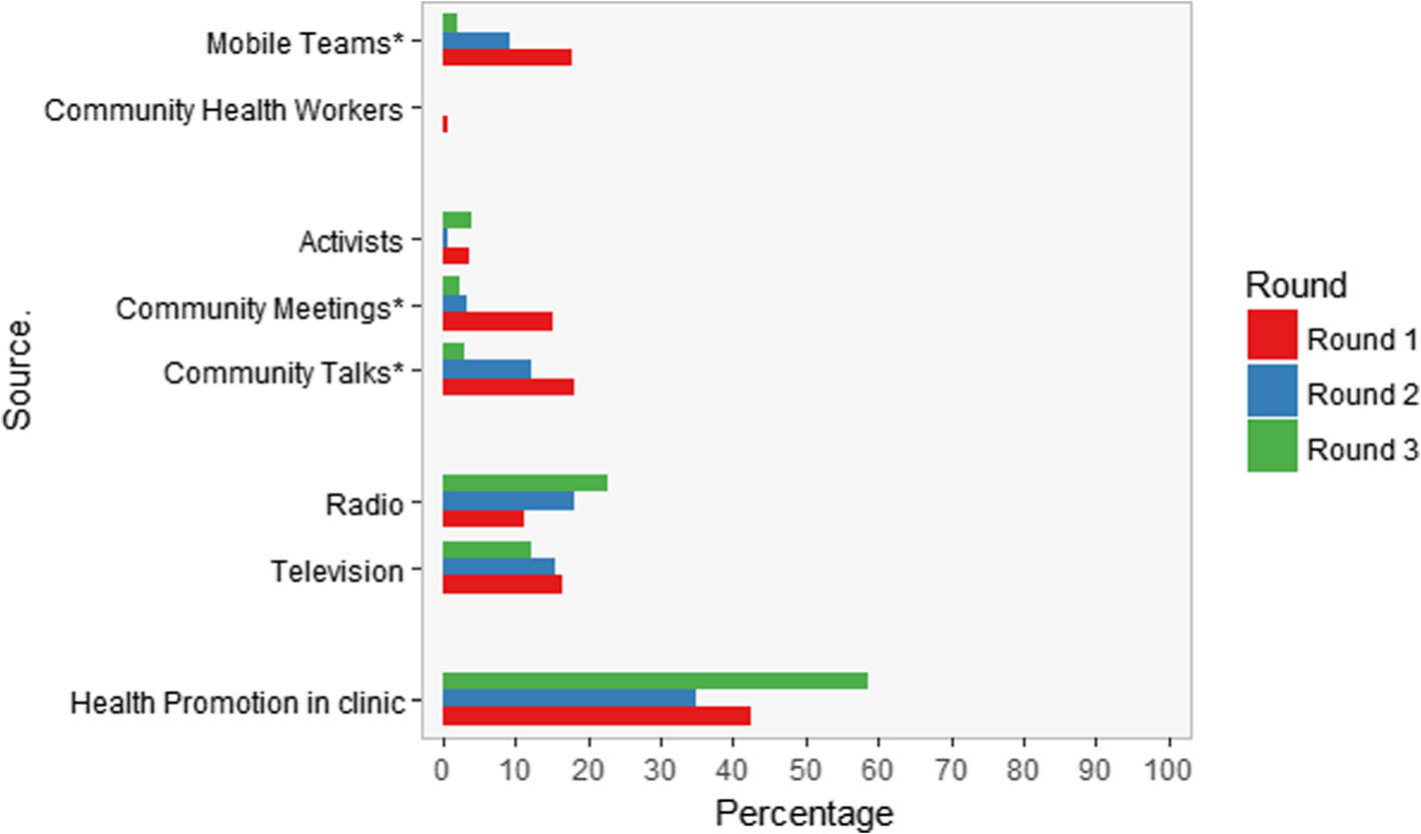
Percentage of female FP users that received information in last 3 months for each source per round. * difference according to round *P* < 0.05

The main source of information was health promotion in the clinic for every round of data collection (Fig. 3). The source of information was stable among the three rounds for: health promotion in the clinic (X^2^ = 11.72, *p*-value = 0.25), television (X^2^ = 0.82, p-value = 0.83), and radio (X^2^ = 7.11, p-value = 0.24). The number of women that received information by community meetings (X^2^ = 18.82, p-value = 0.005), community talks (X^2^ = 12.54, p-value = 0.048) and mobile teams (X^2^ = 15.75, p-value < 0.001) varied significantly according to the round of data collection. Overall, less women were counselled at community level in rounds two and three. For the other sources cell counts were too small to conduct further statistical tests.

We explored which sources of information were associated with higher/lower knowledge regarding LARCs. Taking into account the AICs (Akaike Information Criterion) [37], linear model was built with PC1 as continuous outcome variable and sources of information as dichotomous predictors. PCA loadings ranged from − 4.31 (lowest knowledge) until 3.77 (highest knowledge). Age, marital status and travel time to the facility were all included as covariates but eliminated during model selection as these predictors were not significant and reduced validity of the model. The residuals were normally distributed and the variance was homogenous across the fitted values of the model for each predictor and the response variable. Receiving health promotion in the health facility and information by outreach activities were significant predictors for knowledge regarding LARCs (see Table 5). Women receiving health promotion in the clinic in the last 3 months had significantly lower overall knowledge about LARCs (− 0.864). Women receiving information by outreach activities (by mobile teams or community health workers) had significantly higher overall knowledge about LARCs (+ 0.552).

**Table 5:** Linear Regression Model

	Estimate	Std. Error	t-value	p
Effect:				
Intercept	0.726	0.371	1.960	0.078 .
Received health Promotion in clinic	−0.864	0.265	−3.257	0.008 **
Received info by Community Campaigns	0.087	0.227	0.385	0.708
Received info by Outreach Activities	0.522	0.222	2.354	0.040 *
Received info by Mass Media	0.060	0.184	0.328	0.749

Levels of significance:. = *p* < 0.1; * = *p* < 0.05; ** = *p* < 0.01

## Discussion

This study explored quality of care according to five of the six elements proposed by the framework of Bruce & Jain [22]. The first element, the ‘choice of methods’, refers to having a range of contraceptive methods offered to the clients considering their diverse reproductive, health and behavioral needs. Only by offering a variety of contraceptive options, the health system can respond to the different reproductive, health, and behavioral needs of women [38]. The second element, ‘Information given to clients’, refers to the information provided to users during the consultation, that enables them to choose and use contraception with competence and satisfaction. Both elements are closely linked to each other as they both contribute to the aim of women making a well-informed choice regarding their contraceptive method. The choice of methods will strongly depend on what information women receive during the consultation, what they already know and which methods are available at that moment [39].

Our study showed that providers discussed on average two family planning methods per consultation and that this number did not differ between new users and follow up visits. Especially for new users we would expect that more FP methods are discussed, in order to facilitate a well informed choice among new users. The right of FP clients to receive accurate information and make their own decisions is considered fundamental in sexual and reproductive health and rights (SRHR) [40]. Offering clients information about a variety of methods and letting clients make their own decisions during the consultation is definitely an area in need of improvement in family planning services in rural Mozambique.

LARCs were only discussed with a minority of the FP clients and providers were three times more likely to discuss injections and oral contraceptives. The low level of counseling about LARCs might be explained by certain preferences of the provider or a so called “provider bias” [41]. Previous studies showed that some providers have misconception about LARCs such as the belief that IUDs and implants can only be given to multiparous women [42]. As a consequence, they will not mention the method in consultations with nulliparous women, which will negatively affect women’s ability to choose from all methods. Time constraints might be another reason why providers do not discuss and provide all methods. Mozambique has one of the highest workloads for health providers in Sub-Saharan Africa, with an average of 38 patients a day per provider [43]. Another factor influencing choice may be the availability of methods, which can also be affected by provider bias: providers might stop ordering a method that is not popular [27]. A complementary study focusing on stock outs and the role of providers in the same health centers showed more stock-outs for methods that are less used (female condoms, implants and IUDs) compared to more popular methods such as the injectable and the pill [27].

The third element of the Bruce-Jain framework, ‘technical competence of the provider’, involves providers’ clinical technique, use of protocols, and implementation of aseptic procedures in performing clinical procedures. Technical competence was not assessed in this study. However, it would be interesting to examine in further research whether lack of competence and feeling unconfident is an important barrier to offering LARCs among providers in rural Mozambique. Providers in rural settings do not have many opportunities to learn new techniques or to receive supervision due to time constraints, prioritization of clinical duties, and direct and indirect costs such as transportation, accommodation, and per diems for trainers and supervisors [41].

‘Interpersonal relations’, the fourth element, refer to the degree of empathy, trust, assurance of confidentiality, and sensitivity of providers to meet the client’s needs and expectations. Women reported being very satisfied with the received services, including the way they were treated by the provider. Other researchers examining satisfaction of FP clients in rural Mozambique have reported similar satisfaction rates [44]. The literature indicates that satisfaction is shaped by expectations, and it may be that the women in our study had low expectations regarding FP services which resulted in high satisfaction levels [45].

The fifth element of the framework, follow-up mechanisms, considers how service providers encourage clients on the continuity of use and follow up visits [22, 25]. Almost all women in our study had heard about family planning through different channels in the last 3 months and also during the consultation almost all women were told when to return for a follow-up visit, indicating that providers recognize the importance of continuity and follow up and communicate this effectively to users. Despite the fact that follow up was encouraged, one-third of women were not told where to go in case of problems or emergencies. Given that many women delay seeking care in the hope that symptoms disappear or look for solutions in traditional medicine, providers could stress their availability and responsiveness more strongly to increase the probability that women will seek care in health facilities in case of medical problems [46, 47].

The last component, ‘appropriate constellation of services’, is suitability of family planning services in terms of their location being at convenient place and time and the level of integration with other reproductive and maternal health services. Health examinations were done in very few cases and no difference was found between new users and follow up visits. Also, there were no significant differences between first and follow up visits regarding the number of methods discussed. This suggests family planning consultations are organized as a “one size fits all” approach rather than one that is responsive to the clients’ needs. We would expect standard health examinations for new clients according to global guidelines for family planning consultations [48].

Our research showed that women who received information through outreach activities (mobile teams and community health workers) tended to have better knowledge. A key component of the country’s FP2020 strategy is to engage community health workers (CHWs) and others in sharing information about family planning, and referring community members to sites that offer a wide range of family planning methods [49]. The work of community health workers and mobile teams are part of the national health system in Mozambique [50], but are mainly organized with financial support from bilateral and multilateral cooperation partners and NGOs [51]. This can explain why the number of women counselled by mobile teams was rather small and not stable, as donor funded programs are often restricted in time and resources. The positive results in terms of knowledge found here, strengthen the argument for increased ownership and investment by the national government in these outreach health promotion activities in rural areas, to ensure their continuity and sustainability [52, 53]. Surprisingly, women who had received information about contraceptives in the health center in the last 3 months had lower knowledge regarding LARCs than women who had not. This finding may be explained by different pathways. On the one hand women with low knowledge might visit health centers more often or might be referred to them more often. On the other hand, these findings also suggest that the information that they received at the health center did not improve their knowledge significantly.

Overall, knowledge about LARCs was very poor. Given that the majority of women have not heard of IUDs/implants, more consistent counseling at the health center about LARCs to all women will be essential to ensure that they can make a well-informed choice, reinforced by outreach and community education. Lack of knowledge among women combined with misconceptions is probably an important contributor to the low uptake of LARCs in rural Mozambique.

### Limitations

By examining different components of the Bruce-Jain framework we tried to capture quality of care in a broader sense than using one single item alone. Nevertheless we only used exit interviews with women, and a limitation of patient-reported quality measures is that patients’ memories and assessments of quality may not always be accurate, especially regarding technical quality [54]. A combination of observations and exit interviews, where the technical competence of the provider can also be assessed, would have given a more complete assessment of quality of care in family planning services [55].

Data in this study were collected from clients exiting FP services and need to be interpreted as such. Women using injections and oral contraceptives will visit FP services much more often (overrepresented) than women using LARCs (underrepresented). However, uptake of LARCs among first time users was very low and the same can be seen in national data. To explore the dynamics of contraceptive uptake in the general population, longitudinal studies at household level will be more appropriate [56].

Finally, by using a face-to-face interview as the data collection method we might have induced socially desirable answers from women. This might explain the high satisfaction rates with very little variation in our study. Patient satisfaction is not a clearly defined concept, although it is identified as an important quality outcome indicator to measure quality of care in the literature [20, 57]. We used a standardized questionnaire, which has been one of the most common assessment tools for patient satisfaction studies [57]. However, it might not be the most reliable and valid method for measuring patient satisfaction with FP services in this context and qualitative research can generate more in depth information about women’s experiences with FP services.

Assessments of structures, processes and health outcomes should be carried out to better understand the constellation of services and follow-up mechanisms in rural Mozambique [54]. Follow-up research should also explore knowledge, perceived competence and preferences regarding family planning methods at provider level and the origin of misconceptions by women.

## Conclusion

Despite various efforts, LARC uptake is still very low in two rural districts in Mozambique. Context specific multilevel interventions, beyond training of providers, with long-term follow up are needed to strengthen FP services and LARCs provision in rural Mozambique. Programs at community level to raise awareness and eliminate misconceptions are recommended to increase knowledge and acceptance on the user side. Overall patients are satisfied with the received family planning services but more investments should be made to offer women all methods and related FP information, in order to enable women to make a well informed choice.

## Additional files

Additional file 1: Title: Questionnaire Description: The questionnaire used to assess quality of care (only available Portuguese). (PDF 145 kb)

Additional file 2: Title: PCA analysis knowledge IUDs & implants: Description: The results of the Principal Component Analysis conducted on the knowledge questions about IUDs and implants. (DOCX 55 kb)

## Abbreviations

AIC: Akaike’s Information Criterion
CHWs: Community health workers
DPS: Direcção Provincial de Saúde
FP: Family planning
ICRH: International centre for reproductive health
IUD: Intra-uterine device
LARCs: Long acting reversible contraceptives
LMICs: Low and Middle Income Countries
NGOs: Non-governmental organizations
PCA: Principal component analysis
PSI: Population Services International
SDGs: Sustainable Development Goals
SRHR: Sexual and reproductive health and rights
STIs: Sexually Transmitted Infections
UNFPA: United Nations Population Fund
USAID: United States Agency for International Development
WHO: World Health Organization
