# Supplementary material for: Quality of care in family planning services in rural Mozambique with a focus on long acting reversible contraceptives: a cross-sectional survey
Source: BMC Womens Health. 2018 Dec 12;18:201. doi: 10.1186/s12905-018-0692-z (PMC6291923; doi:10.1186/s12905-018-0692-z)
Supplement: Supplementary file 1 — Title: Questionnaire Description: The questionnaire used to assess quality of care (only available Portuguese). (PDF 145 kb) [file 12905_2018_692_MOESM1_ESM.pdf]

APÊNDICE 3

Início de entrevista (hh/mm): ______

Entrevistas de partida: questionário para mulheres sobre planeamento familiar na província de Maputo, Moçambique

De qual ronda pertence o questionário:

- ◯ Primer
- ◯ Segunda
- ◯ Terceira

Aspectos de identificação do questionário

Nome do inquiridor ______ Data (dia/mês/ano) ______ Número de identificação ______

Nome do supervisor ______ Data (dia/mês/ano) ______

Nome do processador ______ Data (dia/mês/ano) ______

Distrito

- ◯ Marracuene
- ◯ Manhiça

Centro de Saúde

- ◯ Ricatla
- ◯ Mumemo
- ◯ Matalane
- ◯ Mali
- ◯ Eduardo Mondlane
- ◯ Michafutene

Centro de Saúde

- ◯ Maluana
- ◯ Tres de fevereiro
- ◯ Maragra
- ◯ Mwatibwana
- ◯ Malavele
- ◯ Ilha Josina
- ◯ Calanga
- ◯ Taninga
- ◯ Chibucutso

Identificação do respondente

Data de nascimento (dia/mês/ano) ______

Qual é o seu actual estado civil

- ◯ casada
- ◯ união de facto
- ◯ divorciada - separada
- ◯ solteira
- ◯ viúva

Foi antes entrevistado por alguém de de ICRH sobre planeamento familiar?

- ◯ Sim
- ◯ Não

CÓDIGO DO QUESTIONÁRIO :
(Día de nascimento da mulher) ______

A. Serviços de planeamento familiar

1. Recebeu serviços de planeamento familiar?

- ◯ Sim
- ◯ Não => Termine a entrevista

2. Os serviços foram oferecidos de forma espontánea ou teve que solicita-los?

- ◯ Pediu
- ◯ Espontáneamente

3. Que métodos foram discutidos?

- ☐ Nenhum método
- ☐ Implante
- ☐ Esterilização Masculina
- ☐ Pílula
- ☐ DIU
- ☐ Aleitamento exlusivo
- ☐ Preservativo Masculino
- ☐ Injecção
- ☐ Contracepção de emergência
- ☐ Preservativo Feminino
- ☐ Esterilização Feminino
- ☐ Abstinência periódica (Método do Calendário)
- ☐ Método Tradicional
- ☐ Outros Especifique: ____________

4. Recebeu informações suficientes?

- ◯ Sim
- ◯ Não

5. Qual o método que recebeu?

- ☐ Nenhum método
- ☐ DIU
- ☐ Contracepção de emergência
- ☐ Pílula
- ☐ Implante
- ☐ Esterilização Feminina
- ☐ Preservativo Masculino
- ☐ Injecção
- ☐ Esterilização Masculina
- ☐ Outros Especifique: ____________

6. Se recebeu um,foi o método que desejava? Se não recebeu está satisfeita?

- ◯ Sim -> Passar para a pergunta 9
- ◯ Não

7. Se não, qual o método que teria preferido ?

- ☐ Nenhum método
- ☐ Contracepção de emergência
- ☐ Injecção
- ☐ Pílula
- ☐ Esterilização Masculina
- ☐ Implante
- ☐ Preservativo Masculino
- ☐ Esterilização Feminino
- ☐ DIU
- ☐ Preservativo Feminino
- ☐ Outros Especifique: ____________

8.Se não, porquê não recebeu esse método?

- ◯ Não disponível
- ◯ Não sei
- ◯ Não é recomendado
- ◯ Outro motivo

CÓDIGO DO QUESTIONÁRIO : ____________

Para as utentes que receberam um método:
(Para as que não receberam nenhum passa para a pergunta 1 da secção B)

9. Esta satisfeita com o método que lhe foi oferecido?

( ) Sim (Passa para pergunta 11) ( ) Não

10. Se não, por quê?

- [ ] Religião
- [ ] Não é natural
- [ ] Não gosto de usar hormonas
- [ ] Muito pouco tempo de protecção
- [ ] Não é confortável
- [ ] O parceiro não deixa
- [ ] Não conhecia o método
- [ ] Prefiro métodos não-invasivos
- [ ] Não acredito que o método me irá proteger da gravidez
- [ ] Tenho medo dos efeitos colaterais (ganho de peso, naúseas, dor de cabeça, etc)
- [ ] Tenho medo de interferência com a fertilidade
- [ ] Outro: Especifique

11. É a primeira vez que recebe esse método?

( ) Sim ( ) Não

12. Foi-lhe informado sobre como fazer o uso do método?

( ) Sim ( ) Não

13. Durante a consulta o provedor mencionou sobre efeitos colaterais?

( ) Sim ( ) Não (Passa para pergunta 15)

14. Pode mencionar os efeitos colaterais mencionados durante a consulta?

- [ ] Dor de cabeça
- [ ] Dor abdominal
- [ ] Sangramento abundante
- [ ] Sensação de inchaço
- [ ] Mudanças de humor
- [ ] Diminuição de libido
- [ ] Náusea
- [ ] Dôr no peito
- [ ] Pequenas quantidades de sangue entre os períodos
- [ ] Ganho de peso
- [ ] Tonturas
- [ ] Outro Especifique

15.Foi-lhe fornecido algum material sobre o uso do método?

( ) Sim ( ) Não

16. O provedor lhe disse quando voltar para sua próxima consulta?

( ) Sim ( ) Não

17. O provedor informou lhe que poderá ligar ou voltar para US se tiver algum problema com o método?

( ) Sim ( ) Não

CÓDIGO DO QUESTIONÁRIO :

B. Interacções com o profissional de saúde:

1. Foi tratada com respeito e cortesia durante as interacções com o provedor?

◯ Sim ◯ Não

2. Foi convidada a compartilhar informações sensíveis?

◯ Sim ◯ Não -> Passar para a pergunta 4

3. Se Sim: Sentiu que a sua privacidade foi respeitada?

◯ Sim ◯ Não

4. Esteve confortável durante a consulta?

◯ Sim ◯ Não

5. Durante essa visita, o provedor levou-lhe a fazer quaisquer exames ou procedimentos de saúde?

◯ Sim ◯ Não -> Passar para a pergunta 9.

6. Se Sim : Teve privacidade durante os exames e procedimentos com o provedor de saúde?

◯ Sim ◯ Não

7. Se Sim: O provedor explicou-lhe sobre os exames ou procedimentos antes de serem feitos?

◯ Sim ◯ Não -> Passar para a pregunta 9.

8. Se Sim: O provedor explicou-lhe sobre os resultados dos exames de saúde ou procedimentos?

◯ Sim ◯ Não

9. Durante essa visita, teve quaisquer questões que queria fazer?

◯ Sim ◯ Não -> Passar para a secção C

10. Se sim: O provedor deixou-lhe fazer às questões?

◯ Sim ◯ Não -> Passar para a secção C

11. Se sim: O provedor respondeu-lhe às questões a sua satisfação?

◯ Sim ◯ Não

CÓDIGO DO QUESTIONÁRIO : ____________________

C) Acesso

1. O tempo de espera foi aceitável?

( ) Sim ( ) Não

2. As horas em que a unidade sanitária está aberta, são convenientes para si?

( ) Sim -> Passar a pergunta 4. ( ) Não ( ) Não sei -> Passar a pergunta 4

3. Se não, qual seria a hora mais conveniente para si?

[ ] Fins de semana/feriados
[ ] No início da manhã
[ ] A noite
[ ] Durante a hora de almoço
[ ] À tarde
[ ] Não Sei
[ ] Outros Especifique ______

4. Já aconteceu que veio a unidade sanitária e estava fechada?

( ) Sim ( ) Não

5. Quanto tempo em minutos você demorou para chegar aqui hoje?

Tempo (minutos) ______ [ ] Não Sei

6. Qual foi o principal meio de transporte que usou para chegar?

( ) A pé
( ) Ambulância
( ) Transporte Público
( ) Chapa (Transporte semi-colectivo)
( ) Bicicleta ambulância
( ) Transporte à tracção animal
( ) Carro / mota pessoal
( ) Bicicleta
( ) Outro Especifique ______

D) Opçõés sobre a satisfação da utente

1. No geral, diria que ficou satisfeita com a sua visita no dia de hoje ou ficou insatisfeita?

( ) Satisfeia ( ) Insatisfeita

2. Se insatisfeita, por quê?

______

3. Como é que recomendaría esta unidade sanitária à uma amiga que procura os mesmos serviços que a trouxe hoje?

( ) Altamente Recomendado
( ) Não recomendo/ Aconselho a ir a outro lugar
( ) Recomendo Moderamente
( ) Não sei/Indiferente

CÓDIGO DO QUESTIONÁRIO : ______

E) Fontes de informaçao de Planeamanto Familiar

1. Antes da consulta já tinha ouvido falar de PF?

- ( ) Sim
- ( ) Não

2. Nos últimos 3 meses, recebeu alguma informação relacionada ao Planeamento Familiar?

- ( ) Sim
- ( ) Não -> Passar para a pergunta 8

3. Se sim, quais foram as fontes de informação?

- [ ] Palestras na comunidade
- [ ] Rádio
- [ ] Telivisaõ
- [ ] Encontros na comunidade
- [ ] Teatro
- [ ] Brigadas móveis de saúde
- [ ] Activistas
- [ ] APEs
- [ ] Líderes tradicionais
- [ ] Organizações

Se souber dizer, mencione o nome da organização:

- [ ] Palestra na US
- [ ] Outro

Especifique

4. Achou a informação útil?

- ( ) Sim
- ( ) Não

5. A informação era clara?

- ( ) Sim
- ( ) Não

6. A informação recebida mudou a sua opinião?

- ( ) Sim
- ( ) Não -> Passar para a pregunta 8

7. O que mudou?

- [ ] Pensou sobre o assunto e não fez nada
- [ ] Procurou os serviços de PF
- [ ] Falou com parente/amiga sobre PF
- [ ] Falou com o parceiro sobre o PF
- [ ] Outro

Especifique

8. O que se pode melhorar em termos de informação disponibilizada para melhorar a aderência das mulheres ao PF?

CÓDIGO DO QUESTIONÁRIO :

F) Conhecimento e atitude sobre métodos de longa duração (Depo, DIU e implante)

1. Para quem não quer engravidar por um longo período de tempo quais métodos são mais adequados?

- ☐ Depoprovera
- ☐ Implante
- ☐ DIU
- ☐ Preservativo Feminino
- ☐ Microgynon
- ☐ Microlut
- ☐ Preservativo Masculino

2. O implante depois de colocado move-se pelo corpo da mulher

- ○ Não conheço implante
- ○ Não Sei
- ○ Não
- ○ Sim

3. O implante depois de colocado causa dolor

- ○ Não conheço implante
- ○ Não Sei
- ○ Não
- ○ Sim

4. Nos dias após a sua inserção uma mulher que tem implante pode fazer trabalhos pesados (pilar, acarretar água, carregar coisas pesadas, etc.)

- ○ Não conheço Implante
- ○ Não Sei
- ○ Não
- ○ Sim

5. Os fios do DIU podem prender ou "picar" o pénis do homem durante a relação sexual

- ○ Não conheço DIU
- ○ Não
- ○ Não Sei
- ○ Sim

6. Uma mulher que usa o DIU e fica grávida, o bebé nasce com DIU na testa ou na mão

- ○ Não conheço DIU
- ○ Não
- ○ Não Sei
- ○ Sim

CÓDIGO DO QUESTIONÁRIO : ____________________

Fim de entrevista (hh/mm): ____________________

Notas trabalhor de campo

____

Notas digitador

____
