# Supplementary material for: Quality of care in family planning services in rural Mozambique with a focus on long acting reversible contraceptives: a cross-sectional survey
Source: BMC Womens Health. 2018 Dec 12;18:201. doi: 10.1186/s12905-018-0692-z (PMC6291923; doi:10.1186/s12905-018-0692-z)

**PCA ANALYIS KNOWLEDGE IUDs & IMPLANTs**

1. **Importance of components:**

| PC1 PC2 PC3 PC4  Standard deviation 1.7924 0.6277 0.4943 0.3857  Proportion of Variance 0.8032 0.0985 0.0611 0.0372  Cumulative Proportion 0.8032 0.9017 0.9628 1.00000 |
| --- |

1.
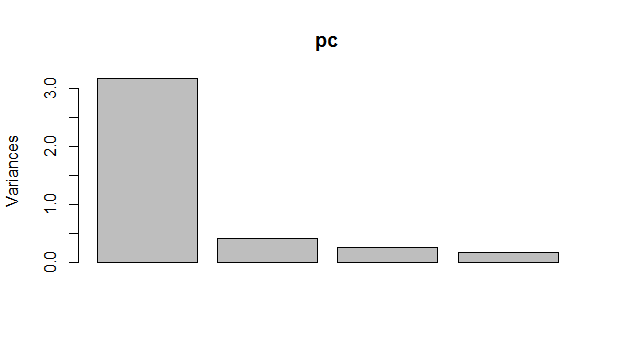
**PCA plot**
2. **Biplot**
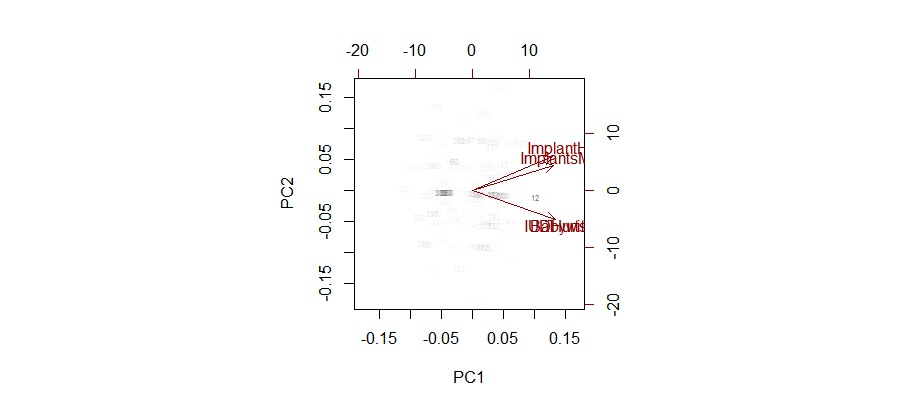

3. **Standard deviation of each of the four PCs, and their rotation (or loadings)**

Standard deviations (1, .., p=4):

[1] 1.7924399 0.6277049 0.4943176 0.3857405

Rotation (n x k) = (4 x 4):

PC1 PC2 PC3 PC4

sydata$F2score 0.4873118 -0.5893082 -0.63406702 -0.1149001

sydata$F3score 0.4963921 -0.4200423 0.74435180 0.1519859

sydata$F5score 0.5092234 0.4784105 0.07563808 -0.7114027

sydata$F6score 0.5067678 0.4973969 -0.19539266 0.6764647

1. Total knowledge score plotted against PC1


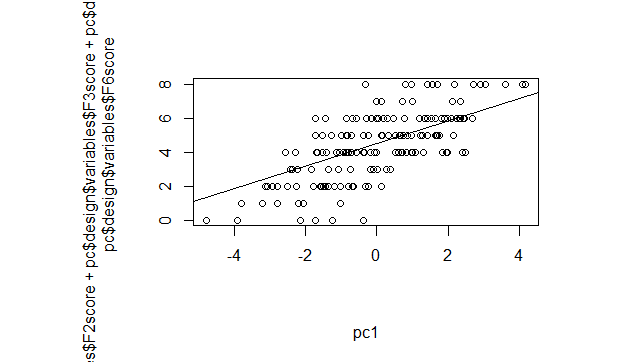

Supplement: Supplementary file 2 — Title: PCA analysis knowledge IUDs & implants: Description: The results of the Principal Component Analysis conducted on the knowledge questions about IUDs and implants. (DOCX 55 kb) [file 12905_2018_692_MOESM2_ESM.docx]
